# Alcohol consumption and leukocyte telomere length

**DOI:** 10.1038/s41598-019-38904-0

**Published:** 2019-02-05

**Authors:** Shalini Dixit, Mary A. Whooley, Eric Vittinghoff, Jason D. Roberts, Susan R. Heckbert, Annette L. Fitzpatrick, Jue Lin, Cindy Leung, Kenneth J. Mukamal, Gregory M. Marcus

**Affiliations:** 1 Department of Medicine, University of California, San Francisco, California USA; 2 Veterans Affairs Medical Center, San Francisco, California USA; 3 Department of Epidemiology and Biostatistics, University of California, San Francisco, California USA; 4 Department of Epidemiology, University of Washington, Seattle, Washington USA; 5 Group Health Research Institute, Group Health Cooperative, Seattle, Washington USA; 6 Department of Global Health and Department of Family Medicine, University of Washington, Seattle, Washington USA; 7 Department of Biochemistry and Biophysics, University of California, San Francisco, California USA; 8 Center for Health and Community, School of Medicine, University of California, San Francisco, California USA; 9 Department of Medicine, Beth Israel Deaconess Medical Center, Harvard Medical School, Boston, Massachusetts USA

## Abstract

The relationship between alcohol consumption and mortality generally exhibits a U-shaped curve. The longevity observed with moderate alcohol consumption may be explained by other confounding factors, and, if such a relationship is present, the mechanism is not well understood. Indeed, the optimal amount of alcohol consumption for health has yet to be determined. Leukocyte telomere length is an emerging quantifiable marker of biological age and health, and a shorter telomere length is a predictor of increased mortality. Because leukocyte telomere length is a quantifiable and objectively measurable biomarker of aging, we sought to identify the amount of alcohol consumption associated with the longest telomere length and least telomere length attrition. Among over 2,000 participants from two distinct cohort studies, we found no pattern of alcohol consumption that was associated with longer telomere length or less telomere length attrition over time. Binge drinking may reduce telomere length. Using telomere length as a marker of age and health, these data fail to demonstrate any benefits of alcohol consumption, even when consumed in moderation.

## Introduction

Telomeres are repetitive DNA sequences (TTAGGG_n_) located at the ends of chromosomes, which protect somatic cells from loss of genomic material during cell division^1^. Though the enzyme telomerase acts to mitigate the amount of telomere shortening with each cell division, there is a progressive loss of nucleotides from the chromosomal ends. Ultimately, telomeres shorten to the point that they cannot maintain chromosomal integrity, leading to cellular senescence and apoptosis^2^. Hence, telomere length is a marker of cellular aging. Increasingly, telomere length (TL) is viewed as a unique biomarker of human age— one that takes into account the cumulative effects of genetic and environmental factors, in addition to chronological age^3^. A shorter TL has been linked to risk factors for cardiovascular disease^4^, myocardial infarction^5^, heart failure^6^, and earlier mortality^7^.

In addition, TL is influenced by common exposures^8–11^ and can lengthen over time, suggesting that perhaps lifestyle choices can not only prevent telomere attrition but even lead to telomere lengthening. Studies on the effects of alcohol on telomere length have been inconclusive and limited to a focus on the harmful effects of alcohol abuse without special attention to the potential beneficial effects of more moderate consumption^12,13^.

While excessive alcohol-use disorders are associated with an increase in cardiovascular disease and mortality^14–17^ moderate alcohol consumption has been associated with a lower risk of coronary disease and reduced mortality^18–20^. Thus, perhaps there is a window of alcohol intake between no consumption and excess consumption that may confer a net health and mortality benefit that can be precisely identified by a longer TL.

## Results

**Figure 1:**
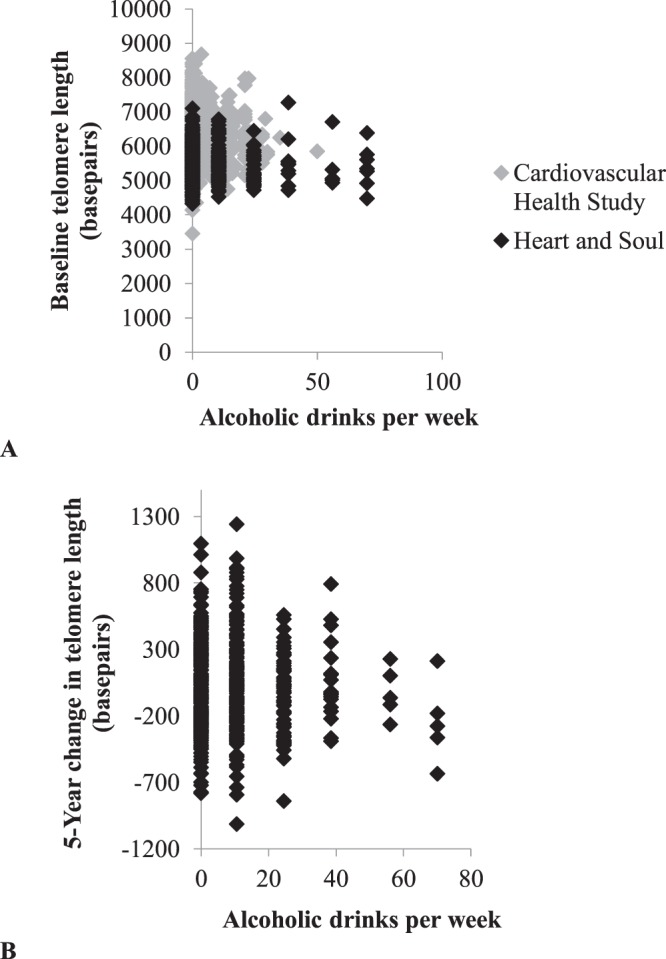
Scatter plots of weekly alcohol consumption and telomere length. Panel A shows average number of drinks per week versus baseline telomere length in the Cardiovascular Health Study (grey, n = 1673) and Heart and Soul (black, n = 948). Panel B shows average number of drinks per week versus 5-year change in telomere length (adjusted for baseline telomere length) among Heart and Soul participants with follow-up data (n = 606).

Characteristics of the participants are presented in Table 1. Heart and Soul participants were younger, predominantly male, more often smokers, and had a greater prevalence of diabetes and cardiovascular comorbidities compared to CHS participants. Alcohol consumers were more likely to be male and white, less likely to have hypertension and heart failure, and had higher levels of the omega-3 fatty acid DHA. Relationships between CRP and omega fatty acids with alcohol consumption and telomere length are shown in Supplementary Table 1a–f. Baseline TL was not different between alcohol consumers and alcohol abstainers in either cohort. 5-year follow-up TL and 5-year change in TL were not different between alcohol consumers and alcohol abstainers in Heart and Soul. Figure 1 shows scatter plots of weekly alcohol consumption versus baseline telomere length (pearson’s *r* = −0.01 for Heart and Soul, pearson’s *r* = −0.04 for CHS) and weekly alcohol consumption versus change in telomere length in Heart and Soul (pearson’s *r* = −0.03), demonstrating no evidence of correlation.

**Table 1:** Characteristics of Heart and Soul and Cardiovascular Health Study Participants by Alcohol Consumption.

	Heart and Soul Study (n = 948)			Cardiovascular Health Study (n = 1673)		
	Alcohol Abstainers (n = 321)	Alcohol Consumers (n = 627)	P Value	Alcohol Abstainers (n = 883)	Alcohol Consumers (n = 790)	P Value
**Demographics**						
Mean age ± SD, *y*	66.1 ± 11.2	67.1 + 10.8	0.202	75.1 ± 5.5	74.5 + 5.0	0.021
Male, *n* (%)	246 (76.6)	527 (84.1)	0.005	314 (35.6)	376 (47.6)	<0.001
**Race**, ***n*** (%)						
White	146 (45.5)	425 (67.9)	<0.001	749 (84.8)	704 (89.1)	0.003
Black	76 (23.7)	79 (12.6)		133 (15.1)	81 (10.3)	
Asian/Hispanic/Other	99 (30.8)	122 (19.5)		1 (0.1)	5 (0.6)	
**Health & Habits**						
Mean BMI ± SD, *kg/m*^2^	28.7 ± 6.0	28.3 ± 5.0	0.309	27.1 ± 5.0	26.7 ± 4.3	0.046
Mean waist-to-hip ratio ± SD	0.95 ± 0.08	0.96 ± 0.08	0.520	0.95 ± 0.08	0.95 ± 0.07	0.608
Median kilocalories of physical activity (IQR)	—	—	—	769 (245–1710)	945 (303–2221)	<0.001
**Smoking status**, ***n*** (%)						
Current smoker	69 (21.6)	120 (19.1)	0.377	89 (10.3)	106 (13.7)	<0.001
Non-smoker	251 (78.4)	507 (80.9)		449 (51.8)	241 (31.1)	
Former smoker	—	—		329 (38.0)	429 (55.3)	
**Usual packs per day during smoking years**						
<1/2 pack per day	41 (18.6)	93 (21.1)	0.816	—	—	—
<1 pack per day	65 (29.6)	131 (29.7)				
<2 packs per day	78 (35.5)	142 (32.2)				
≥2 packs per day	36 (16.4)	75 (17.0)				
Median drinks per week (IQR)	0 (0–0)	11 (11-11)	<0.001	0 (0-0)	1.3 (0.3–7.0)	<0.001
**Medical History**						
Diabetes mellitus, *n* (%)	108 (33.6)	143 (22.8)	<0.001	88 (12.7)	97 (14.2)	0.402
Hypertension, *n* (%)	244 (76.0)	427 (68.1)	0.011	538 (61.3)	425 (53.9)	0.002
Coronary artery disease, n (%)	—	—	—	214 (24.2)	175 (22.2)	0.314
Myocardial infarction, *n* (%)	173 (54.3)	334 (53.5)	0.837	22 (2.5)	17 (2.2)	0.646
Heart failure, *n* (%)	85 (26.5)	118 (18.9)	0.007	85 (9.6)	39 (4.9)	<0.001
Stroke, *n* (%)	—	—	—	21 (2.4)	11 (1.4)	0.142
Liver disease, *n* (%)	—	—	—	7 (0.8)	5 (0.7)	0.709
**Inflammatory Markers**						
Median C-reactive protein (IQR), *mg/L*	2.5 (1.0–6.0)	2.1 (0.87–4.35)	0.087	3.0 (1.3–6.8)	2.8 (1.4–6.3)	0.225
Median interleukin-6 (IQR), *mg/dL*	2.8 (1.8–4.3)	2.5 (1.5–4.0)	0.085	2.9 (2.0–4.4)	2.8 (1.9–4.3)	0.067
Mean fibrinogen ± SD, *mg/dL*	398.3 ± 87.5	390.4 ± 91.4	0.205	341.0 ± 75.4	324.8 ± 70.4	<0.001
**Omega-3 Fatty Acid Plasma Levels**						
Median DHA (IQR), *% total fatty acids*	2.85 (2.21–3.66)	3.04 (2.34–4.14)	0.004	2.76 (2.28–3.38)	2.93 (2.33–3.64)	0.008
Median EPA (IQR), *% total fatty acids*	0.52 (0.39–0.74)	0.68 (0.47–1.13)	<0.001	0.81 (0.71–0.93)	0.82 (0.73–0.93)	0.106
**Telomere Length**						
Baseline mean leukocyte telomere length^a^ ± SD, *kilobase pairs*	5.48 ± 0.55	5.42 ± 0.51	0.091	6.34 ± 0.63	6.31 ± 0.60	0.239
5-year follow-up mean leukocyte telomere length^b^ ± SD, *kilobase pairs*	5.30 ± 0.35	5.28 ± 0.36	0.614	—	—	—
Median 5-year absolute change in telomere length (IQR)^b^, *basepairs*	−268 (−588–133)	−133 (−488–177)	0.065	—	N/A	N/A

BMI = body mass index; DHA = docosahexaenoic acid; EPA = eicosapentaenoic acid; IQR = interquartile range; SD = standard deviation. Values are reported as mean ± SD, median (IQR), or number (percentage). ^a^Measured on blood specimen from 1992–93 for CHS participants. ^b^For follow-up, n = 606 participants.

### Linear relationships between alcohol consumption and leukocyte telomere length

No linear association between alcohol consumption and baseline TL in Heart and Soul in either unadjusted or adjusted analyses was observed (Table 2). In CHS, more alcohol consumption was statistically significantly associated with a shorter TL in unadjusted analysis and after adjustment for age, but the relationship was no longer statistically significant after further adjustment (Table 2).

**Table 2:** Association of Alcohol Consumption with Baseline Telomere Length.

**Heart and Soul (n = 948)**		
**Model** ^**a**^	**Beta Coefficient (95% CI)**	**P Value**
Unadjusted	−0.04 (−2.58, 2.50)	0.98
Age-adjusted	−1.00 (−3.52, 1.53)	0.44
Model 1	−1.38 (−3.91, 1.16)	0.29
Model 2	−1.09 (−4.06, 1.89)	0.66
Model 3	−1.34 (−4.33, 1.66)	0.38
Model 4	−1.64 (−4.67, 1.39)	0.29
**Cardiovascular Health Study (n = 1673)**		
**Model** ^**b**^	**Beta Coefficient (95% CI)**	**P Value**
Unadjusted	−7.53 (−13.56, −1.49)	0.015
Age-adjusted	−7.43 (−13.35, −1.51)	0.014
Model 1	−3.25 (−9.20, 2.70)	0.29
Model 2	−2.40 (−8.47, 3.67)	0.44
Model 3	−3.24 (−9.99, 3.51)	0.35
Model 4	−1.46 (−9.11, 6.18)	0.71

Alcohol consumption was modeled continuously in drinks per week; telomere length was measured in basepairs.

^a^Age-adjusted model was adjusted for baseline (year 1) age. Model 1 was adjusted for age, sex, and race. Model 2 = Model 1 + BMI, waist-hip ratio, smoking status, and number of pack years. Model 3 = Model 2 + diabetes, hypertension, previous myocardial infarction, and heart failure. Model 4 = Model 3 + C-reactive protein, interleukin-6, fibrinogen, docosahexaenoic acid, and eicosapentaenoic acid.

^b^Age-adjusted model was adjusted for baseline (year 5) age. Model 1 was adjusted for age, sex, and race. Model 2 = Model 1 + BMI, waist-hip ratio, kilocalories of physical activity, and smoking status. Model 3 = Model 2 + diabetes, hypertension, coronary artery disease, prior myocardial infarction, heart failure, prior stroke, and liver disease. Model 4 = Model 3 + C-reactive protein, interleukin-6, fibrinogen, docosahexaenoic acid, and eicosapentaenoic acid.

In the longitudinal analyses in Heart and Soul, we found no linear association between alcohol consumption and 5-year change in telomere length (Table 3).

**Table 3:** Association of Alcohol Consumption with Change in Leukocyte Telomere Length Over 5 Years.

Model^a^	Beta Coefficient (95% CI)	P Value
**Heart and Soul (n = 606)**		
Unadjusted	−0.93 (−3.07, 1.21)	0.40
Age-adjusted	−1.41 (−3.47, 0.65)	0.18
Model 1	−0.60 (−2.66, 1.47)	0.57
Model 2	−0.20 (−2.57, 2.17)	0.87
Model 3	−0.36 (−2.75, 2.03)	0.77
Model 4	−0.41 (−2.84, 2.02)	0.74

Alcohol consumption was modeled continuously in drinks per week.

Change in telomere length in basepairs was defined as the residual of the regression of 5-year change in telomere length on baseline telomere length.

^a^Age-adjusted model was adjusted for baseline (year 1) age. Model 1 was adjusted for age, sex, and race. Model 2 = Model 1 + BMI, waist-hip ratio, smoking status, and number of pack years. Model 3 = Model 2 + diabetes, hypertension, previous myocardial infarction, and heart failure. Model 4 = Model 3 + C-reactive protein, interleukin-6, fibrinogen, docosahexaenoic acid, and eicosapentaenoic acid.

### Relationships between categories of alcohol consumption and leukocyte telomere length

We found no consistent associations between categorical amounts of alcohol consumption and change in telomere length (Supplementary Table 2). However, in Heart and Soul, consuming >14 drinks per week compared to none was associated with approximately 119 fewer basepairs (fully adjusted β coefficient = −118.15, 95% CI −234.05 to −2.25, p 0.046). Tests for trends across levels of consumption failed to reveal any statistically significant linear or non-linear trends.

### Analyses of binge drinking and “ideal” drinking

**Figure 2:**
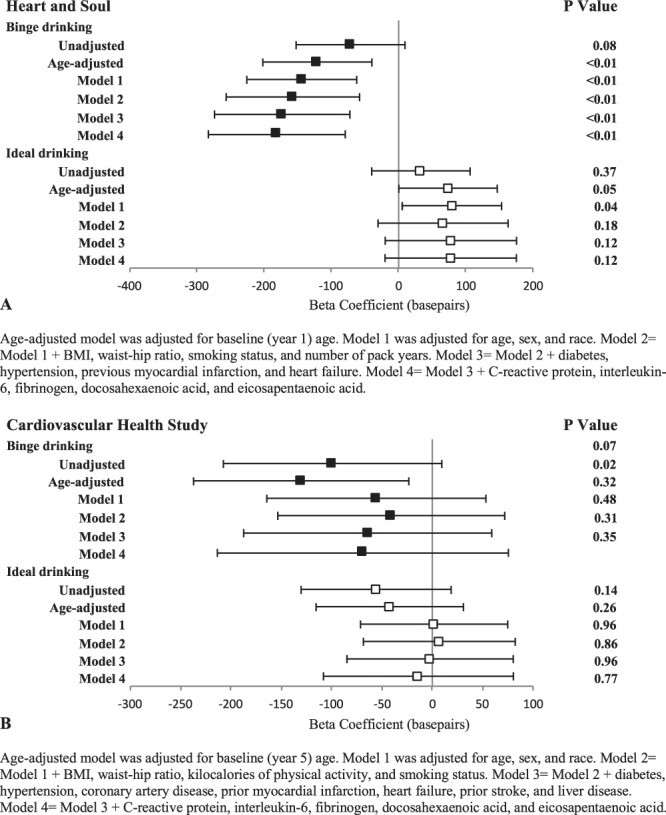
Associations of binge drinking (black square) and ideal drinking (white square) with baseline telomere length. See methods for classification of binge and ideal drinking. Y error bars denote 95% confidence intervals.

In Heart and Soul, binge drinking was associated with a statistically significantly shorter TL in adjusted analyses (Fig. 2). While the point estimates similarly favored a shorter TL among binge drinkers in CHS, none of the models with the exception of age-adjusted reached statistical significance. Ideal drinking was generally associated with longer TLs in Heart and Soul, only reaching statistical significance in Model 1 and losing statistical significant in all other models (Fig. 2). No relationship with ideal drinking was observed in CHS.

**Figure 3:**
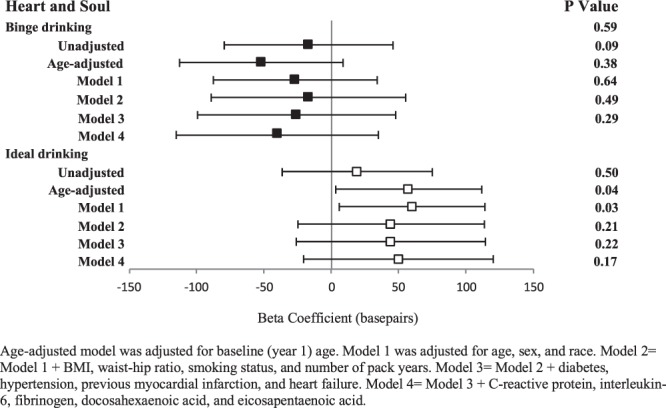
Associations of binge drinking (black square) and ideal drinking (white square) with change in telomere length in Heart and Soul. See methods for classification of binge and ideal drinking; comparison group for each analysis is non-binge drinkers and non-ideal drinkers, respectively. Change in telomere length is adjusted for baseline telomere length. Y error bars denote 95% confidence intervals.

Although no statistically significant findings were observed, the point estimates generally revealed a more negative change in 5-year TL in Heart and Soul with binge drinking. Ideal drinking was associated with point estimates supporting an increase in TL. This reached statistical significance in the age-adjusted model and Model 1 alone, but no other models exhibited statistical significance after additional potential confounders were included (Fig. 3).

### Alcohol type and telomere length

In CHS, no statistically significant relationships between amount of weekly wine, beer, or hard liquor consumption and TL were observed after multivariable adjustment.

## Discussion

In an investigation of over 900 Heart and Soul participants and over 1,600 CHS participants, we found no consistent relationship between alcohol consumption and either baseline TL or 5-year change in TL. We observed a statistically significant relationship between binge drinking and shorter telomere length after comprehensive multivariable adjustment in Heart and Soul. In no circumstance was there evidence of a relationship between any amount of alcohol consumption, including moderate or “ideal” consumption, and TL lengthening after taking potential confounders into account.

Telomere length has emerged as a unique marker of aging that integrates the effects of genetic, environmental, behavior, and psychosocial factors with biological aging^3,8–10^. Consistently, shorter TL has been linked with increased risk of age-associated diseases^4–6,21,22^ and more importantly with cardiovascular and overall mortality^1,7^.

In recent years, research has shifted towards examining the rate of telomere change as a predictor of longevity and mortality, with the rationale that change in telomere length compared to a single TL assessment would account for any advantage conferred by a longer TL at baseline^11^. The hope is that identifying modifiable exposures that enhance TL maintenance and lengthening could help determine prevention strategies against age-associated morbidities and premature mortality.

Alcohol consumption is often described as bearing a J-shaped relationship with mortality as a result of its combination of both beneficial and harmful effects^23,24^. While low levels of alcohol intake (1-2 drinks per day for women and 2–4 drinks per day for men) have been inversely associated with coronary heart disease^18^ and overall mortality^20^, higher levels of alcohol intake are associated with increased risk of cirrhosis, certain cancers, trauma and various adverse cardiovascular outcomes^17,25^. Given the mortality benefits of moderate alcohol consumption, we hypothesized that there may be a window of alcohol intake associated with longer TL and greater increase in TL over time. However, while we cannot exclude a small benefit to “ideal” drinking or harm from binge drinking, our findings do not support any relationship between any amount of alcohol consumption and a longer TL.

Though the relationship between alcohol consumption and TL has been previously studied, the results have been mixed. In a cohort study of 500 middle-aged Finnish men, greater midlife alcohol consumption was significantly associated with having a shorter TL later in life, while current alcohol consumption in old age was not^26^. While interesting, the findings of this study seem prone to unmeasured confounding given that this was an observational study measuring TL over 25 years after ascertainment of alcohol consumption, and they only adjusted for age, baseline smoking, BMI, cholesterol, and perceived fitness. In contrast, in an analysis of 4,000 men and women in the Copenhagen City Heart Study, researchers were unable to identify a relationship between alcohol consumption and baseline TL or 10-year change in TL, in accordance with our findings^12^. While their study featured a large sample size and follow-up TL measurements, they relied on self-reported heavy drinking as the predictor variable without regard to number of drinks or frequency of consumption. In yet another study of over 450 healthy volunteers aged 20–50 years, no association between alcohol consumption and TL was observed; however, they did not investigate type of alcohol consumed and lacked longitudinal TL data. Finally, all of these studies solely sought to examine whether more alcohol was associated with shorter TL and did not attempt to identify an ideal pattern of consumption that may be beneficial or specific patterns that may be harmful.

Among the strengths of our current study are the detailed assessments of demographic, behavioral, medical, and biochemical covariates in two cohorts and the longitudinal study design with change in TL data. Our study population draws from two very different cohorts with distinct age ranges, racial composition, comorbidities, and drinking patterns. To our knowledge, we are the first to examine the relationship between alcohol type and TL, binge drinking and TL, and the first to seek to investigate an ideal pattern of consumption.

Several limitations of our study must be acknowledged. As this was an observational study, we cannot make definitive statements regarding causality. Although we adjusted for several carefully assessed potential confounding variables, there is the possibility of residual confounding by measured covariates or confounding by unmeasured covariates. We used self-reported data as a measure of participants’ alcohol consumption. While self-report has been validated as an accurate, reproducible method of evaluating alcohol consumption^27,28^, it is possible that this introduced some misclassification of exposure data into our analysis. Furthermore, we did not have data regarding past alcohol consumption in Heart and Soul, as the AUDIT-C refers to alcohol use over the past year, and only have a limited assessment of past alcohol consumption in CHS. We also lacked longitudinal data on alcohol consumption and therefore assumed that participants’ baseline drinking was reflective of their typical pattern of drinking and constant over time. In addition, we only measured telomere length in leukocytes, and it is possible that alcohol has varying effects on different cell types. However, TL is known to correlate highly with TL in cells from other tissues, such as the vascular wall^29,30^, and, as shown by our group, myocardial samples^31^. In addition, TL was measured in older age in both cohorts and, therefore, restricted to survivors. It is possible that individuals with extremely detrimental drinking patterns and/or extremely short TL died prematurely and hence were not included in this study. Finally, we did not measure telomere length in specific leukocyte subgroups; future research may determine whether other markers of aging and immunosenescence in particular (such as by utilizing CD8 telomere length) might provide additional insights regarding beneficial or harmful effects of alcohol^32^.

In summary, we found no evidence that any amount or type of alcohol consumption was associated with a longer TL or an increase in TL over time. Although not reproducible across both cohorts, binge drinking remained associated with a shorter telomere length even after comprehensive adjustments for potential confounders in the Heart and Soul Study. Future research addressing binge drinking and telomere length, including telomere length after cessation of binge drinking, is needed.

## Methods

We assessed the relationship between alcohol consumption and TL in two longitudinal cohort studies, the Heart and Soul Study and the Cardiovascular Health Study (CHS). The Heart and Soul study was selected as the primary cohort given its repeated telomere measurements.

The research protocol for Heart and Soul was approved by the institutional review boards at the University of California, San Francisco, and the San Francisco VA Medical Center. CHS was approved by the institutional review boards at the University of California, Davis, the Johns Hopkins University, the Wake Forest University School of Medicine, and the University of Pittsburgh. All participants in both studies provided written informed consent, and all research was performed in accordance with relevant guidelines and regulations.

### The Heart and Soul Study

#### Study design

Eligible participants were recruited from outpatient clinics in the San Francisco Bay Area of California if they had a history of myocardial infarction, angiographic evidence of at least 50% stenosis by area in at least one coronary artery, evidence of stress-induced ischemia, or history of coronary revascularization^33^. Individuals were excluded if they had a history of myocardial infarction in the past six months, reported being unable to walk one block, or were planning to relocate out of the area within three years.

#### Study cohort

Between 2000 and 2002, 1024 participants enrolled in the study. Between 2005 and 2007, 667 (80%) of the eligible 829 participants completed a 5-year follow-up examination. Participants without baseline TL measurements or alcohol measurements were excluded (n = 76) leaving 948 participants for the baseline analyses. Of the 667 participants at 5-year follow-up, 61 were excluded because they did not have both baseline and follow-up TL measurements, and an additional 2 were excluded because they did not have baseline alcohol data.

#### Assessment of alcohol consumption

During the baseline examination, participants completed the Alcohol Use Disorders Identification Test (AUDIT-C) (Supplementary Table 3), a validated self-report alcohol screening test that quantifies regular alcohol use and identifies patients with hazardous patterns of alcohol consumption or active alcohol use disorders^34,35^.

#### Covariate ascertainment

Baseline demographics, age, sex, race, ethnicity, smoking status, and comorbidities including diabetes mellitus, hypertension, heart failure, and history of previous myocardial infarction were determined by questionnaire. Weight and height were measured, and body mass index (BMI) was calculated as weight in kilograms divided by height in meters squared. Waist and hip circumferences were measured with a flexible plastic measure to the nearest 0.1 cm.

Please see the Supplementary Methods 1 for details regarding measurement of C-reactive protein (CRP), interleukin-6 (IL-6), fibrinogen, and omega-3 fatty acids docosahexaenoic acid (DHA) and eicosapentaenoic acid (EPA).

#### Measurement of telomere length

Using DNA isolated from peripheral blood leukocytes, relative mean TL was measured from DNA by a quantitative polymerase chain reaction (qPCR) assay that compares mean telomere repeat sequence copy number (T) to a reference single-copy gene copy number (S) in each sample as previously described and validated^36^. Standard curves were derived from serially diluted reference DNA^36–38^. The T/S ratio was determined from the mean quantity of reference DNA found to match with each experimental sample for the copy number of the targeted template^10^. All PCRs were carried out on a Roche Lightcycler 480 real-time PCR machine (Roche Applied Science, Indianapolis, Indiana).

The T/S ratio at baseline and at follow-up were measured in duplicate and averaged for each participant. When the duplicate and the initial values differed by more than 7%, another the sample was run, and the 2 closest values were averaged. We observed an inter-assay coefficient of variation for telomere length measurement of 3.7% and an intra-assay coefficient of variation of 2.5%^10^. As in previous analyses, we converted the T/S ratio to base pairs using the formula: base pairs = 3274 + 2413*(T/S)^39,40^.

### The Cardiovascular Health Study

#### Study design

In short, 5,201 individuals 65 years or older were recruited between 1989 and 1990 from a random sample of Medicare beneficiaries by four academic centers (Johns Hopkins University, Wake Forest University, University of Pittsburgh, and University of California, Davis)^41–43^. An additional 687 black patients were recruited between 1992 and 1993. All participants had a medical history, physical exam, laboratory testing, and 12-lead electrocardiogram (ECG) performed. Participants were followed with annual clinic visits and semiannual telephone contact for ten years, with telephone contact continued every 6 months thereafter.

#### Study cohort

Our analysis was restricted to the subset of 1675 participants randomly selected for TL measurement from participants who completed the 1992–93 annual clinic examination (considered baseline for the current analysis). Participants with missing alcohol data at the 1992–93 exam (n = 2) were excluded.

#### Assessment of alcohol consumption

Participants reported their usual frequency of consumption of alcoholic beverages (daily, weekly, monthly, yearly, or rarely/never) and the usual number of 12-ounce cans or bottles of beer, 6-ounce glasses of wine, and shots of liquor that they drank on each occasion. Participants also reported whether they had changed their pattern of consumption during the past 5 years and whether they had ever regularly consumed five or more drinks daily^44^.

#### Covariate ascertainment

Self-identified race, sex, income, and education level were obtained at baseline, while the rest of the covariates included in this analysis were obtained in 1992–93. BMI was calculated from weight and height obtained at the yearly clinic visit. Physical activity in kilocalories was calculated from the physical activity questionnaire. Medical comorbidities including diabetes mellitus, hypertension, coronary artery disease (CAD), heart failure, and liver disease were assessed through a combination of clinic measurements, self-report, and medical record review. Events including myocardial infarction and stroke were individually adjudicated.

Please see the Supplementary Methods 2 for details regarding measurements of CRP, IL-6, fibrinogen, DHA and EPA fatty acids.

#### Measurement of telomere length

Using DNA isolated from peripheral leukocytes, TL was measured using Southern blot analysis of terminal restriction fragment lengths and reported in kilobases^4,45,46^. Telomere measurements were performed in duplicate (with each sample analyzed on two different gels on two different occasions), and the mean was used for statistical analyses. The Pearson’s Correlation Coefficient for duplicates was 0.96, with an average coefficient of variation for paired sets of 1.7%. The laboratory conducting the TL measurements was blinded to all characteristics of participants.

#### Categorization of alcohol consumption (both cohorts)

For Heart and Soul, binge drinking was defined as present when participants reported drinking ≥6 drinks on at least one occasion in the past year. Binge drinking in CHS was defined as drinking >5 drinks most days.

For the purposes of the study, we defined “ideal” drinking as moderate alcohol consumption. Specifically, for Heart and Soul, ideal drinking was defined as 1–2 drinks per day and no binge drinking (the AUDIT-C did not differentiate between 1 and 2 drinks per day). For CHS, ideal drinking was defined as 1–7 drinks per week and no binge drinking for female participants and 1–14 drinks per week and no binge drinking for male participants. The associations between the number of drinks of each alcohol type per week and TL were examined in CHS.

#### Statistical methods

Continuous variables with normal distribution are presented as means + standard deviation (SD) and were compared using the Student’s t-test. Non-normally distributed continuous variables are presented as medians with interquartile ranges (IQRs) and were compared using the Kruskal–Wallis test. The association between categorical variables was determined using the chi-square test.

Linear regression was used to examine the cross-sectional association between alcohol intake and TL in both cohorts. Multivariable adjustment was made for potential demographic, medical, and biochemical confounders based on published associations and biological plausibility^4,9,47–49^. In addition to an age-adjusted model, 4 multivariate models were developed: Model 1included age, sex, and race; Model 2 included the Model 1 covariates plus BMI, waist-hip ratio, physical activity, smoking status, and number of pack years; Model 3 included Model 2 covariates plus diabetes, hypertension, coronary artery disease, prior myocardial infarction, heart failure, prior stroke, and liver disease; and Model 4 included Model 3 covariates plus inflammatory markers (CRP, IL-6, fibrinogen) and omega-3 fatty acid levels (DHA, EPA).

Given that previous studies have demonstrated a strong relationship between baseline TL and subsequent change in TL^39^, we calculated a change score that is adjusted for baseline TL to use as our main outcome.

Models were assessed for linearity using residual versus predicted plots and component plus residual plots. Normality was assessed using Q-Q plots of residuals. Constant variance across groups of alcohol consumption was verified. Influential points were assessed using boxplots of dfbeta statistics, which identified five potential outliers. When the models were re-fit excluding those potential outliers, our overall conclusions remained unchanged.

Data were analyzed using Stata 13 (StataCorp, College Station, TX, USA). A two-tailed p < 0.05 was considered statistically significant.

## Supplementary information

Supplementary Information

## Data Availability

The data underlying our work can be obtained through the following mechanisms. For Heart and Soul, the data are owned by the Department of Veterans Affairs and cannot be shared publicly due to the data set containing protected health information. Requests for data may be sent to Mary Whooley, MD (San Francisco VA Health Care System) at mary.whooley@va.gov or mary.whooley@ucsf.edu. For CHS, interested investigators can contact CHS to become a new investigator/ collaborator. Details about the procedures for data request can be found on the following website: https://chs-nhlbi.org/NewInvest. In addition, most CHS data can be also obtained from BioLINCC, a repository maintained by the National Heart, Lung,and Blood Institute. The BioLINCC website (https://biolincc.nhlbi.nih.gov/) includes detailed information about the available data and the process to obtain such data. Any interested researcher could obtain the de-identified, minimal datasets needed to replicate or reprove the study findings pending ethical approval through the aforementioned mechanisms.
