# Supplementary material for: Alcohol consumption and leukocyte telomere length
Source: Sci Rep. 2019 Feb 5;9:1404. doi: 10.1038/s41598-019-38904-0 (PMC6363724; doi:10.1038/s41598-019-38904-0)
Supplement: Supplementary file 1 — Supplementary Information [file 41598_2019_38904_MOESM1_ESM.docx]

**Alcohol consumption and leukocyte telomere length**

Shalini Dixit^1^, Mary A. Whooley^1,2,3^, Eric Vittinghoff^3^, Jason D. Roberts^1^, Susan R. Heckbert^4,5^, Annette L. Fitzpatrick^6^, Jue Lin^7^, Cindy Leung^8^, Kenneth J. Mukamal^9^, Gregory M. Marcus^1^

^1^Department of Medicine, University of California, San Francisco, California, USA. ^2^Veterans Affairs Medical Center, San Francisco, California, USA. ^3^Department of Epidemiology and Biostatistics, University of California, San Francisco, California, USA. ^4^Department of Epidemiology, University of Washington, Seattle, Washington, USA. ^5^Group Health Research Institute, Group Health Cooperative, Seattle, Washington, USA. ^6^Department of Global Health and Department of Family Medicine, University of Washington, Seattle, Washington, USA. ^7^Department of Biochemistry and Biophysics, University of California, San Francisco, California, USA. ^8^Center for Health and Community, School of Medicine, University of California, San Francisco, California, USA. ^9^Department of Medicine, Beth Israel Deaconess Medical Center, Harvard Medical School, Boston, Massachusetts, USA.

***Corresponding Author:**

Gregory M. Marcus, MD, MAS

505 Parnassus Ave, M-1180B, Box 0124

San Francisco, CA 94143-0124

Phone: (415) 476-5706, Fax: (415) 353-9190

Email: marcusg@medicine.ucsf.edu

**Supplementary Table 1a.** Association of Alcohol Consumption with C-Reactive Protein

|  | **Beta Coefficient (95% CI)** | **P Value** |
| --- | --- | --- |
| **Heart and Soul (n=948)** |  |  |
| Unadjusted | -0.02 (-0.06, 0.03) | 0.45 |
| Adjusted | -0.02 (-0.05, 0.02) | 0.41 |
| **CHS (n=1673)** |  |  |
| Unadjusted | -0.07 (-0.19, 0.04) | 0.20 |
| Adjusted | 0.06 (-0.04, 0.15) | 0.22 |

Alcohol consumption was modeled continuously in drinks per week; C-reactive protein was measured in mg/L. The adjusted model (Model 4) was adjusted for age, sex, race, BMI, waist-hip ratio, smoking status, number of pack years, diabetes, hypertension, previous myocardial infarction, heart failure, C-reactive protein, interleukin-6, fibrinogen, docosahexaenoic acid, and eicosapentaenoic acid.

**Supplementary Table 1b.** Association of Alcohol Consumption with Docosahexaenoic Acid Levels

|  | **Beta Coefficient (95% CI)** | **P Value** |
| --- | --- | --- |
| **Heart and Soul (n=948)** |  |  |
| Unadjusted | -0.001 (-0.001, 0.001) | 0.06 |
| Adjusted | -0.001 (-0.001, -0.001) | 0.05 |
| **CHS (n=1673)** |  |  |
| Unadjusted | -0.01 (-0.02, 0.004) | 0.24 |
| Adjusted | -0.004 (-0.02, 0.007) | 0.48 |

Alcohol consumption was modeled continuously in drinks per week; docosahexaenoic acid (DHA) levels were represented as percentage of total omega-3 fatty acid plasma levels. The adjusted model (Model 4) was adjusted for age, sex, race, BMI, waist-hip ratio, smoking status, number of pack years, diabetes, hypertension, previous myocardial infarction, heart failure, C-reactive protein, interleukin-6, fibrinogen, and eicosapentaenoic acid.

**Supplementary Table 1c.** Association of Alcohol Consumption with Eicosapentaenoic Acid Levels

|  | **Beta Coefficient (95% CI)** | **P Value** |
| --- | --- | --- |
| **Heart and Soul (n=948)** |  |  |
| Unadjusted | -0.001 (-0.001, 0.001) | 0.69 |
| Adjusted | 0.001 (-0.001, 0.001) | 0.21 |
| **CHS (n=1673)** |  |  |
| Unadjusted | 0.001 (-0.001, 0.002) | 0.57 |
| Adjusted | 0.001 (-0.001, 0.003) | 0.48 |

Alcohol consumption was modeled continuously in drinks per week; eicosapentaenoic acid (EPA) levels were represented as percentage of total omega-3 fatty acid plasma levels. The adjusted model (Model 4) was adjusted for age, sex, race, BMI, waist-hip ratio, smoking status, number of pack years, diabetes, hypertension, previous myocardial infarction, heart failure, C-reactive protein, interleukin-6, fibrinogen, and docosahexaenoic acid.

**Supplementary Table 1d.** Association of C-Reactive Protein with Telomere Length

|  | **Beta Coefficient (95% CI)** | **P Value** |
| --- | --- | --- |
| **Heart and Soul (n=948)** |  |  |
| Unadjusted | -0.27 (-6.59, 6.05) | 0.93 |
| Adjusted | -3.23 (-15.2, 8.72) | 0.60 |
| **CHS (n=1673)** |  |  |
| Unadjusted | -0.21 (-2.83, 2.42) | 0.88 |
| Adjusted | -4.04 (-1.09, 9.17) | 0.12 |

C-reactive protein was measured in mg/L; telomere length was measured in basepairs. The adjusted model (Model 4) was adjusted for age, sex, race, BMI, waist-hip ratio, smoking status, number of pack years, diabetes, hypertension, previous myocardial infarction, heart failure, C-reactive protein, interleukin-6, fibrinogen, docosahexaenoic acid, and eicosapentaenoic acid.

**Supplementary Table 1e.** Association of Docosahexaenoic Acid Levels with Telomere Length

|  | **Beta Coefficient (95% CI)** | **P Value** |
| --- | --- | --- |
| **Heart and Soul (n=948)** |  |  |
| Unadjusted | -3225 (-6322, -128.6) | 0.04 |
| Adjusted | -4893 (-11615, 1829.3) | 0.15 |
| **CHS (n=1673)** |  |  |
| Unadjusted | 39.8 (6.01, 73.61) | 0.02 |
| Adjusted | 33.3 (-8.22, 74.86) | 0.12 |

Docosahexaenoic acid (DHA) levels were represented as percentage of total omega-3 fatty acid plasma levels; telomere length was measured in basepairs. The adjusted model (Model 4) was adjusted for age, sex, race, BMI, waist-hip ratio, smoking status, number of pack years, diabetes, hypertension, previous myocardial infarction, heart failure, C-reactive protein, interleukin-6, fibrinogen, docosahexaenoic acid, and eicosapentaenoic acid.

**Supplementary Table 1f.** Association of Eicosapentaenoic Acid Levels with Telomere Length

|  | **Beta Coefficient (95% CI)** | **P Value** |
| --- | --- | --- |
| **Heart and Soul (n=948)** |  |  |
| Unadjusted | -2813 (-7506, 1880) | 0.24 |
| Adjusted | 7038 (-3166, 17243) | 0.18 |
| **CHS (n=1673)** |  |  |
| Unadjusted | -50.4 (-242.1, 141.4) | 0.61 |
| Adjusted | 12.8 (-213.4, 239.0) | 0.91 |

Eicosapentaenoic acid (EPA) levels were represented as percentage of total omega-3 fatty acid plasma levels; telomere length was measured in basepairs. The adjusted model (Model 4) was adjusted for age, sex, race, BMI, waist-hip ratio, smoking status, number of pack years, diabetes, hypertension, previous myocardial infarction, heart failure, C-reactive protein, interleukin-6, fibrinogen, docosahexaenoic acid, and eicosapentaenoic acid.

**Supplementary Table 2.** Adjusted associations of categories of weekly alcohol consumption with baseline telomere length.

| **Heart and Soul (n=948)** | | |
| --- | --- | --- |
| **Weekly Consumption^a^** | **Beta Coefficient (95% CI)** | **P Value** |
| None | (reference) |  |
| 7 to <14 drinks | -5.68 (-102.62, 91.25) | 0.91 |
| >14 drinks | -118.15 (-234.05, -2.25) | 0.046 |

| **Cardiovascular Health Study (n=1673)** | | |
| --- | --- | --- |
| **Weekly Consumption^b^** | **Beta Coefficient (95% CI)** | **P Value** |
| None | (reference) |  |
| Former | -166.10 (-279.21, -53.00) | 0.004 |
| <1 drink | -24.02 (-135.02, 86.98) | 0.67 |
| 1 to <7 drinks | -101.05 (-222.15, 20.05) | 0.10 |
| 7 to <14 drinks | 1.79 (-168.35, 171.93) | 0.98 |
| > 14 drinks | -100.33 (-256.54, 55.87) | 0.21 |

Alcohol consumption was modeled as a categorical variable according to reported weekly consumption and telomere length measured in basepairs. Abstainers (“None” group) were used as the reference for the regression analyses. In both tables, analyses were adjusted for: age, sex, race, BMI, waist-hip ratio, smoking status, number of pack years, diabetes, hypertension, previous myocardial infarction, heart failure, C-reactive protein, interleukin-6, fibrinogen, docosahexaenoic acid, and eicosapentaenoic acid.

^a^Due to a lack of data on past drinking in Heart and Soul, the none category includes all participants abstaining from alcohol at baseline (including former drinkers).

^b^Former drinkers were defined as those with a previous drinking history who were abstaining prior to and at the time of TL measurement (1992-1993).

**Supplementary Table 3.** Alcohol Use Disorders Identification Test (AUDIT-C) Questions and Answer Choices

| 1. How often did you have a drink containing alcohol in the past year? Consider a drink to be a can or bottle of beer, a glass of wine, a wine cooler, one cocktail or a shot of hard liquor (like scotch, gin, or vodka).   - Never - Monthly or less - 2–4 times per month - 2–3 times a week - 4–5 times a week - ≥6 times a week |
| --- |
| 2. How many drinks did you have on a typical day when you were drinking in the past year?   - 0 drinks - 1–2 drinks - 3–4 drinks - 5–6 drinks - 7–9 drinks - ≥10 drinks |
| 3. How often did you have 6 or more drinks on one occasion in the past year?   - Never - Less than monthly - Monthly - Weekly - Daily or almost daily |

**Supplementary Methods 1**

**Additional covariate ascertainment (Heart and Soul Study)**

Fasting venous blood samples were obtained at the baseline visit and again at the follow-up visit. C-reactive protein (CRP) was measured using the Roche Integra assay (Roche Diagnostics, Indianapolis, Indiana) or, due to a change in the laboratory, the Beckman Extended Range assay (Beckman, Galway, Ireland). A Quantikine High Sensitivity Immunoassay kit (R&D Systems, Minneapolis, Minnesota) was used to measure interleukin-6 (IL-6) serum concentration. Fibrinogen was measured using the Clauss assay (30). Levels of the marine omega-3 fatty acids docosahexaenoic acid (DHA) and eicosapentaenoic acid (EPA) were measured in fasting whole blood. Fatty acid methyl esters generated by treatment with boron trifluoride-methanol were analyzed by capillary gas chromatography (GC2010 [Shimadzu Corp, Columbia, Maryland] equipped with a 100-m SP2560 column [Supelco; Bellefonte, Pennsylvania]) and identified by comparison with a known standard (GLC-727; Nuchek Prep, Elysian, Minnesota). Blood levels of DHA and EPA are expressed as a percentage of total fatty acid methyl esters (10).

**Supplementary Methods 2**

**Additional covariate ascertainment (Cardiovascular Health Study)**

Measurements of C reactive protein, IL-6, fibrinogen, DHA and EPA fatty acids were all performed on blood samples from the 1992-93 visit. Blood sample acquisition in addition to analytical and quality assurance methods in CHS have been previously reported (37). Briefly, participants underwent phlebotomy in the morning after an overnight fast; all measurements of inflammatory biomarkers and fatty acids were performed on blood stored in the central CHS laboratory (38). CRP was measured using stored plasma and a high-sensitivity enzyme-linked immunosorbent assay (ELISA) (39) developed and validated in CHS laboratories (analytical coefficient of variation 5.1%) (40). IL-6 was measured from stored sera using a commercial ELISA kit (Quantikine IL-6, R&D Systems, Minneapolis, MN; coefficient of variation 6.3%)(41). Fibrinogen was measured using a modified Von Clauss method (coefficient of variation 2.9%) (36). Methods for the assessment of plasma phospholipid FAs have been described previously in detail (42, 43). In short, plasma lipids were extracted according to Folch et al (44), the phospholipid fractions were isolated by thin-layer chromatography, then the phospholipid FA moieties were transmethylated and separated by gas chromatography (100 m × 0.25 mm silica column, model 6890; Agilent Technologies Inc)(45). Blood levels of DHA and EPA are expressed as a percentage of total phospholipid FAs.
